# Distinct Bacterial and Fungal Communities Colonizing Waste Plastic Films Buried for More Than 20 Years in Four Landfill Sites in Korea

**DOI:** 10.4014/jmb.2206.06021

**Published:** 2022-11-17

**Authors:** Joon-hui Chung, Jehyeong Yeon, Hoon Je Seong, Si-Hyun An, Da-Yeon Kim, Younggun Yoon, Hang-Yeon Weon, Jeong Jun Kim, Jae-Hyung Ahn

**Affiliations:** 1 Agricultural Microbiology Division, National Institute of Agricultural Sciences, Rural Development Administration (RDA), Wanju-gun, Jeollabuk-do 55365, Republic of Korea; 2 Macrogen Inc., Seoul 06221, Republic of Korea; 3 College of Environmental and Bioresource Sciences, Jeonbuk National University, Iksan 54596, Republic of Korea

**Keywords:** Biodegradation, landfill, microbial community, polyethylene

## Abstract

Plastic pollution has been recognized as a serious environmental problem, and microbial degradation of plastics is a potential, environmentally friendly solution to this. Here, we analyzed and compared microbial communities on waste plastic films (WPFs) buried for long periods at four landfill sites with those in nearby soils to identify microbes with the potential to degrade plastics. Fourier-transform infrared spectroscopy spectra of these WPFs showed that most were polyethylene and had signs of oxidation, such as carbon-carbon double bonds, carbon-oxygen single bonds, or hydrogen-oxygen single bonds, but the presence of carbonyl groups was rare. The species richness and diversity of the bacterial and fungal communities on the films were generally lower than those in nearby soils. Principal coordinate analysis of the bacterial and fungal communities showed that their overall structures were determined by their geographical locations; however, the microbial communities on the films were generally different from those in the soils. For the pulled data from the four landfill sites, the relative abundances of *Bradyrhizobiaceae*, *Pseudarthrobacter*, *Myxococcales*, *Sphingomonas*, and *Spartobacteria* were higher on films than in soils at the bacterial genus level. At the species level, operational taxonomic units classified as *Bradyrhizobiaceae* and *Pseudarthrobacter* in bacteria and *Mortierella* in fungi were enriched on the films. PICRUSt analysis showed that the predicted functions related to amino acid and carbohydrate metabolism and xenobiotic degradation were more abundant on films than in soils. These results suggest that specific microbial groups were enriched on the WPFs and may be involved in plastic degradation.

## Introduction

Petroleum-based plastics are widely used in all industries due to their flexibility and structural stability. However, as the non-degradable traits of plastics have harmful effects on the natural environment [1], the biodegradation of plastics has emerged as an eco-friendly disposal method that has been actively studied. Examples of notable achievements in this field include the isolation of polyethylene terephthalate (PET)-degrading *Ideonella sakaiensis* [2], as well as improvement of the degradative capacity of PETases by protein engineering [3, 4]. However, conclusive evidence for the biodegradation of polyethylene (PE) and polypropylene (PP), which together account for most of the plastic used in packaging [5], is still lacking despite a large amount of literature reporting biodegradation of these materials [6-8].

PE is a highly non-degradable plastic due to its structural simplicity and stability [9]. PE films have been extensively used for agriculture, and their proper disposal is an important environmental issue. In addition, PE mulching films and greenhouse films are representative agricultural wastes and can be a source of microplastics and agrochemicals leaking into the surrounding environment [10-12].

Plastics are known to comprise 7-20% of municipal solid waste (MSW) [13, 14], and thus MSW landfills are seen as ideal locations for plastic biodegradation [15-18]. So far, research has mostly focused on the isolation of plastic-degrading microorganisms from landfills, and only a few studies have investigated the microbial communities on waste plastics to identify these plastic-specific microorganisms.

Moreover, many studies have reported that biofilms on the surface of plastics in marine environments harbor distinct microbial communities and have suggested the possibility of microbial biodegradation of plastics [19], but there are relatively few studies on terrestrial environments. Puglisi E *et al*. [20] and MacLean J *et al*. [21] investigated the bacterial communities on waste plastics in abandoned landfills and a plastic recycling factory, and reported *Bacillus* and *Tychonema* as the most enriched bacterial genera on plastics, respectively. Wright RJ *et al*.[22] identified significantly more abundant taxonomic groups in the “plastisphere” in marine, freshwater, and terrestrial environments at each taxonomic level based on data from 2,229 samples within 35 studies. However, it is still unclear whether there is a microbial community specific to plastic debris, such as microplastics, in terrestrial environments [23].

In the present study, we collected and analyzed the bacterial and fungal communities on WPFs buried for more than 20 years in four landfill sites and compared them with those in the nearby soils under the assumption that there would be microbial groups specific to plastics across all four sites. Furthermore, we sought clues regarding microorganisms capable of degrading plastics, particularly polyethylene.

## Materials and Methods

### Landfill Site Description and Sample Collection

Three WPF samples and three soil samples within a 1-m radius of the films were collected from each of the four landfill sites in Korea, the details of which are presented in Table 1 and Fig. S1. Only highly weathered WPF samples were collected (Fig. S2). The wastes were estimated to have been buried for more than 26, 20, and 40 years in the respective cities of Gunsan (GS), Jeongeup (JE), and Jeju (JJ), and 28 years in the county of Sunchang (SC) based on the closing years of the landfill sites (GS and SC), the landfill manager (JE), and the date of manufacture printed on the WPFs (JJ). The samples were placed in sterilized plastic bags and transported to the laboratory in an icebox. All of the collected samples from the four landfill sites were also used for FT-IR and community analysis.

### Characterization of WPFs by FTIR Analysis

The functional WPF groups were characterized by Fourier-transform infrared (FTIR) spectroscopy. Samples were cleaned to remove biomass contamination according to the recommendations of Sandt C *et al*. [24]: they were cut into 1.5 × 1.5 cm^2^ pieces, rinsed with deionized water, and incubated in deionized water at 60°C for 1 h to remove proteins. The samples were then incubated in 50% ethanol at 60°C for 1 h to remove fats, followed by a sonication bath in a 1 M sodium hydroxide solution for 5 min to remove organic acids. Sonication was repeated five times, and the samples were finally rinsed with deionized water and dried at 60°C for 2 h. Attenuated total reflection (ATR) spectra of five different areas on individual samples of film were obtained using a platinum diamond on an Alpha-II FTIR spectrometer (Bruker, Germany) with a resolution of 4 cm^-1^ and a scanning range from 4,000 cm^-1^ to 400 cm^-1^. The FTIR spectra were analyzed using OPUS 8.5 software, and the values obtained from five areas on the film samples were averaged and represented in a graph. An additive-free LDPE film (GoodFellow, UK) was used as a control.

### Analysis of Bacterial and Fungal Communities on WPFs and in Nearby Soils

To isolate metagenomic DNA, soil samples were meshed through a 2-mm sieve to remove the large particles, and the WPFs were washed once with distilled water to remove the soil particles. All samples were subjected to DNA isolation using the FastDNA Spin Kit for Soil (MP Biomedicals, USA).

The structures of the bacterial and fungal communities in the WPFs and soils were analyzed by sequencing the PCR amplicons of bacterial 16S rRNA genes and fungal ITS regions, which were amplified using primers Bakt_341F/Bakt_805R [25] and ITS3/ITS4 [26], respectively. Paired-end sequencing was performed using an Illumina MiSeq sequencer (Illumina, USA) according to the Illumina 16S metagenomics library preparation protocol [27] by Macrogene, Inc. (Korea).

The bioinformatics pipeline used USEARCH (version 9.1.13) [28] and MOTHUR programs (version 1.39.5)[29]. Using the USEARCH commands, overlapping paired-end sequence reads were merged (fastq_mergepairs), and reads shorter than 300 bp were removed (fastq_filter). Then, reads were clustered into operational taxonomic units (OTUs) at a cutoff of 0.03, and chimera sequences were removed (cluster_otus). The resulting OTU table was transformed into a shared file using customized Perl scripts for use in the MOTHUR program. Using the MOTHUR commands, taxonomic assignment was carried out (classify.seqs) based on the Ribosomal Database Project database (RDP version 18) [30] for bacteria and the UNITE database (version 10) [31] for fungi (iters = 1,000 and cutoff = 60).

After the read number in each sample was normalized to that of the sample with the smallest reads (sub.sample), Good’s coverage, species richness indices (Chao1 and ACE), and diversity indices (Shannon and inverse Simpson) were calculated (summary.seqs).

The bacterial reads were aligned using the SINA aligner (version 1.1) [32] and SILVA 16S rRNA database (SSURef-138.1) [33]. A phylogenetic tree was constructed using FastTree [34], and unweighted and weighted Fast UniFrac distances between samples [35] were calculated with the resulting tree and the abundance data using the MOTHUR command (unifrac). Principal coordinate analysis (PCoA) was performed based on the distance measures using the MOTHUR command. PCoA of the fungal communities was performed based on the unweighted (Jaccard) and weighted (Bray-Curtis) dissimilarity indices calculated from the OTU tables (shared file). Non-metric Multidimensional Scaling (NMDS) analysis of the microbial communities was carried out based on the Sørenson dissimilarity index using the MOTHUR command (dist.shared(calc=sorest)).

The 16S rRNA gene sequences of the bacterial OTUs significantly enriched on the waste plastic films and those of the closely related sequences obtained from GenBank were aligned, and a neighbor-joining tree was constructed based only on the aligned segments (about 450 bp) using the Molecular Evolutionary Genetics Analysis (MEGA) software program, version 6 [36].

### Co-Occurrence Network Analysis for the Microbial Communities of Landfill Plastisphere

We investigated the inter-domain co-occurrence network within the microbial communities on the WPFs and in the nearby soils using the extended version of SPIEC-EASI (Sparse Inverse Covariance Estimation for Ecological Association Inference) [37] and the merged OTU table of 16S rRNA genes and ITS regions. In addition, each bacterial and fungal community network was constructed through the original version of SPIEC-EASI [38]. OTUs occurring in less than 50% of the samples were filtered for the common OTUs among samples, and the Meinshausen–Bühlmann neighborhood method was used for all network analyses. The network was then visualized by *ggnet* package in R [39].

### Functional Prediction of Film-Related Bacterial Communities Using PICRUSt

Phylogenetic Investigation of Communities by Reconstruction of Unobserved States (PICRUSt) [40] was carried out using the web-based Galaxy-PICRUSt platform (https://huttenhower.sph.harvard.edu/galaxy/). The predicted functional profiles were categorized by KEGG pathway [41] and those with higher abundances on the films than in the soils were identified.

### Statistical Analysis

Significant differences among the diversity indices or the relative abundances of microbial taxa were analyzed using Student’s *t*-test or one-way analysis of variance (ANOVA) followed by Duncan’s test. Data were tested for the ANOVA assumptions of independence by Durbin-Watson test, normality of residuals by Shapiro-Wilk test, and homogeneity of variance by Bartlett test. *p*-values < 0.05 were considered significant. All analyses were performed using R Studio (version 1.4.1717; R software, version 4.1.1) [42].

### Data Availability

Quality-screened sequences were deposited in the NCBI SRA database under BioProject accession numbers PRJNA682528 and PRJNA770487.

## Results

### Analysis of WPFs Based on FTIR Spectra

**Fig. 1:**
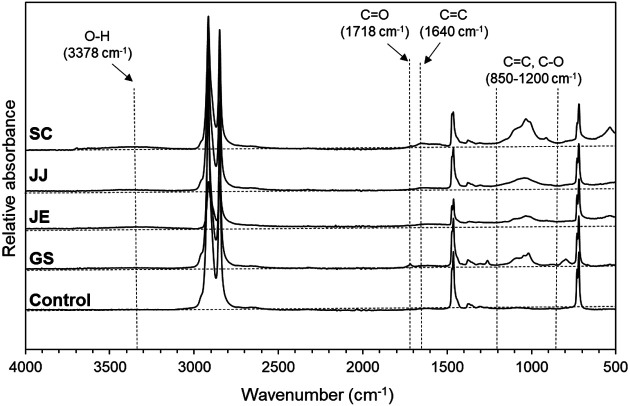
Infrared spectra of the representative WPFs collected from the four landfill sites. GS, JE, JJ, and SC indicate the landfill sites from which the plastic films were collected. Seemingly most oxidized PE film was represented among the three replicates per site.

Images of waste plastic films collected from the four landfill sites are shown in Fig. S2. The FTIR spectra of the films showed that most of them were PE because they had the typical absorption bands of PE (Fig. 1) [43], although two of them showed a significantly different spectral pattern from that of PE (GS-2 and SC-2 in Fig. S3).

Most of the waste films had three broad adsorption regions indicating the oxidation of the films (Fig. 1): one ranging from 850 to 1,200 cm^-1^, corresponding to the absorptions of the C=C of vinyl, vinylene, or vinylidene groups, as well as the C-O of peroxides, hydroperoxides, secondary or tertiary alcohols, or esters; one around 1,640 cm^-1^ corresponding to the absorption of C=C; and one around 3,378 cm^-1^ corresponding to the OH vibrations of alcohols [44, 45]. In contrast, only one sample collected from GS had a small adsorption band (1,718 cm^-1^) corresponding to carbonyl groups (C=O). This result is comparable with that observed in the waste PE films collected from an abandoned landfill [20] and contrasts with those observed in the LDPE films exposed to sunlight, in which bands corresponding to carbonyl groups appeared extensively [44, 46, 47]. This indicates that the rate of oxidation of polyethylene films in landfills is slower than that of the films exposed to sunlight. This is attributed to the deficiency of UV radiation and oxygen in landfills, which are the most important factors for the weathering of polyolefins [48].

### Microbial Species Richness and Diversity on WPFs

The bacterial and fungal community structures were analyzed based on the bacterial 16S rRNA genes and fungal ITS regions amplified from the total DNA samples extracted from the WPFs and nearby soils at the four landfill sites.

Good’s coverage reached over 0.932 in bacterial communities and 0.995 in fungal communities, indicating that the number of reads was sufficient to cover most of the bacterial and fungal diversities (Table 2). The species richness indices (the number of OTUs, Chao1, and ACE) indicated that the number of bacterial and fungal species decreased on the films compared to those in the nearby soils in GS, JE, and JJ, and vice versa in SC, although significant differences between the soils and films (*p* < 0.05) were not observed in all of the samples (Table 2). Diversity indices – Shannon and inverse Simpson – were also lower on the films than in the soils, except for the bacterial community in GS and the fungal community in SC. This result suggests that the WPFs generally decreased the species richness and diversity of the bacterial and fungal communities surrounding them. Two previous studies on the bacterial communities on waste plastics showed similar trends [20, 21], although the evenness was higher on the films than in the soils in one of the studies [20].

### Structures of Bacterial Communities on WPFs

**Fig. 2:**
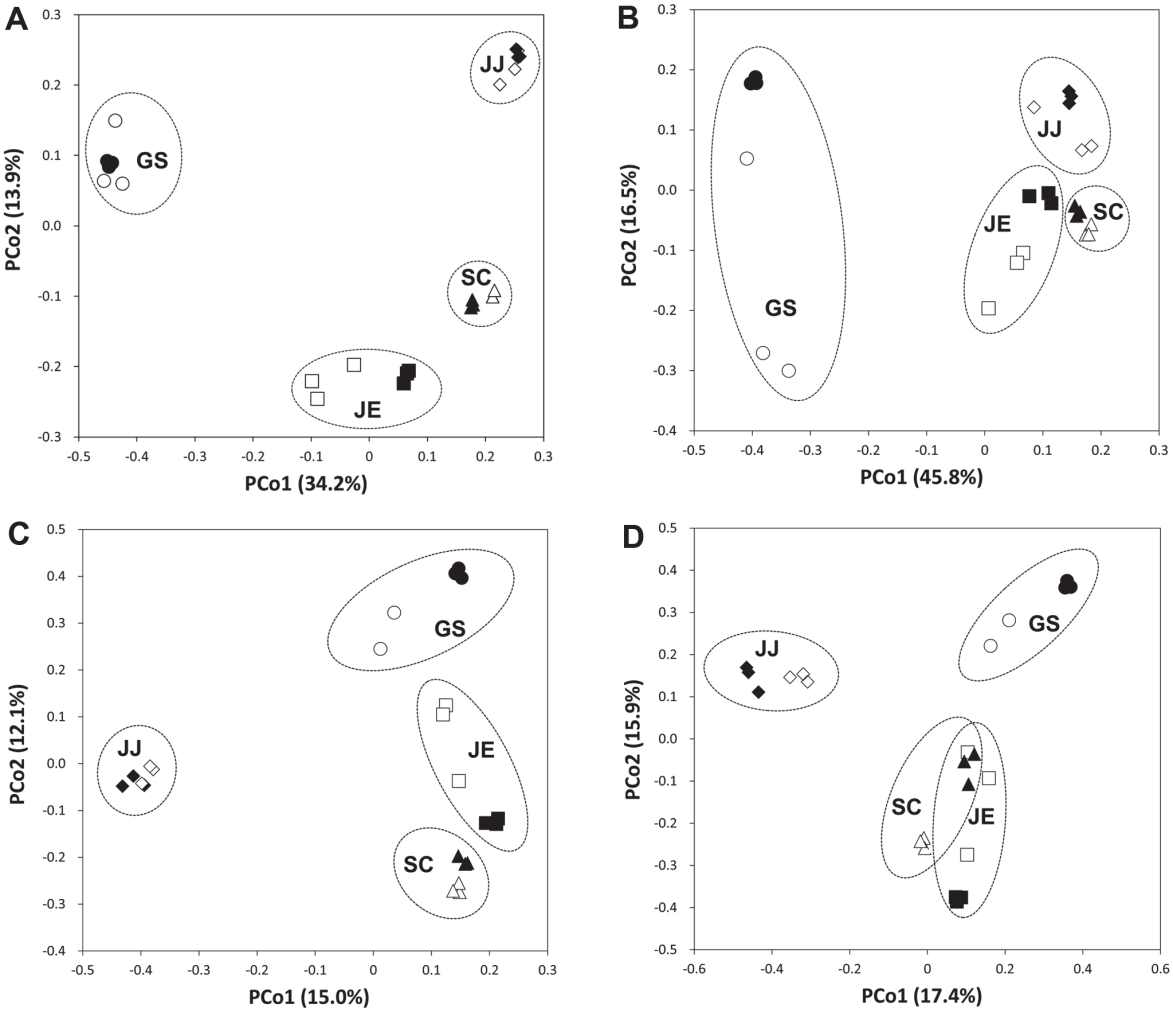
Principal coordinate analysis (PCoA) of the microbial communities. Bacterial (**A** and **B**) and fungal (**C** and **D**) communities on WPFs (open symbols) and in nearby soils (closed symbols) were analyzed with the unweighted (**A** and **C**) and weighted (**B** and **D**) Fast UniFrac metric.

PCoA of the bacterial communities based on the unweighted and weighted Fast UniFrac metric showed that they were grouped mainly by the site from which they were sampled (Figs. 2A and 2B). It also showed that the bacterial communities on the WPFs were different from those in the nearby soils because those on the WPFs were generally separated from those in the nearby soils in the PCoA plots. These results indicate that the overall structure of the bacterial community was determined by its geographical origin, which influences the soil physicochemical properties and climatic conditions, and that the bacterial communities on the WPFs were different from those in the nearby soils. NMDS of the bacterial communities also showed similar trends to those observed in PCoA plots (Fig. S4A). Similar results were also observed for bacterial communities on waste plastics in other works [20, 21]. In addition, the PCoA plots showed that the bacterial communities from GS were relatively different from those of the others because principal coordinate 1 separated the two groups. Methane-oxidizing (*Methylobacter* and *Methylomicrobium*) and sulfur-oxidizing (*Thiobacillus* and *Thioalkalispira*) bacterial groups accounted for the dominant genera at GS, but not at the others (Fig. S5), indicating that anaerobic respiration, such as methanogenesis, might be occurring more at GS than at the other sites, as observed by the previous study [49], resulting in bacterial communities different from those at the other sites. In addition, the PCoA plots showed that the difference in community structures between the soils and films was relatively larger at GS and JE than at JJ and SC. This might be due to the length of time during which the waste plastics were buried (Table 1), as Puglisi E *et al*. [20] suggested that the microbial community on plastic approaches that in the soils as its degradation proceeds.

To determine which taxonomic groups were enriched on the films, we identified those with higher abundances on the films than in the soils at the phylum, genus, and species (OTU) levels. In this process, we arbitrarily defined film-enriched groups as those with higher abundances on the films than in the soils, and with abundances higher than 1.5% (at the phylum level), 1.0% (at the genus level), and 0.5% (at the OTU level) on the films for the pulled data of the four sites.

**Fig. 3:**
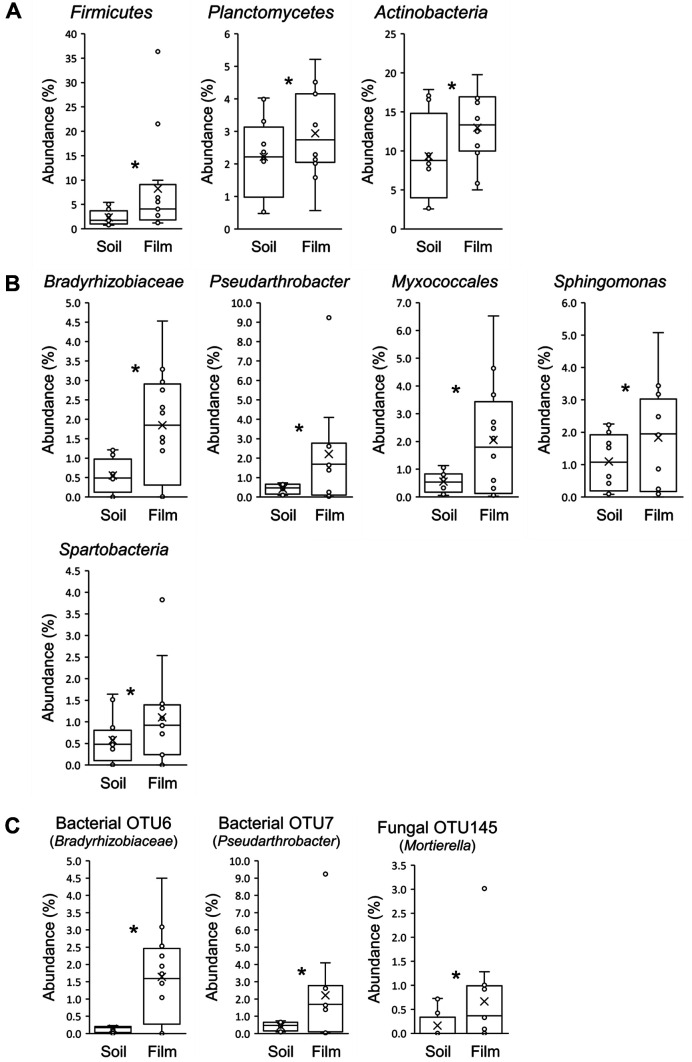
Box plots of the bacterial and fungal taxonomical groups enriched on the waste plastic films. The top, middle, and bottom lines of each box represent the 25th, 50th, and 75th percentiles and the whiskers represent the maximum and minimum excluding outliers. The abundance values (*n* = 12) and their averages were denoted by ○ and ×, respectively. Each taxonomic group was selected with higher relative abundances on the films than in the soils and with abundances higher than 1.5% (at the phylum level), 1.0% (at the genus level), and 0.5% (at the OTU level) on the films. The asterisks (*) indicate that the abundance on the film is significantly higher than that in the soil (*p* < 0.05).

At the phylum level *Firmicutes* and *Planctomycetes* were enriched on the films (Fig. 3A) and had higher abundances on the film than in the soil at each of the four sites, although significant difference was observed only for *Firmicutes* in SC (Fig. S6). *Actinobacteria* were also enriched on the films (Fig. 3A) and were more abundant on the film than in the soil in GS, JJ, and SC (Fig. S6C). Among the three phyla enriched on plastic films, *Actinobacteria* were reported as one of the significantly enriched bacterial phyla in terrestrial plastispheres based on a meta-analysis of 49 samples retrieved from previous studies [22].

At the genus level, *Bradyrhizobiaceae*, *Pseudarthrobacter*, *Myxococcales*, *Sphingomonas*, and *Spartobacteria* were enriched on the films (Fig. 3B), and *Bradyrhizobiaceae* and *Spartobacteria* had higher abundances on the film than in the soil at each of the four sites, whereas *Pseudarthrobacter*, *Myxococcales*, and *Sphingomonas* were higher in JE, JJ, and SC (Fig. S7). Besides these five genera, *Mycobacterium* was enriched on the films in GS, JJ, and SC, *Polyangiaceae* in JE and SC, and *Anaerolineaceae* and *Methylobacter* in GS (Fig. S7), indicating that some bacterial groups enriched on waste plastics may be site-specific.

At the species (OTU) level, OTU6 (*Bradyrhizobiaceae*) and OTU7 (*Pseudarthrobacter*) were enriched on the films (Fig. 3C). The abundance of these two OTUs was higher on the films than in the soils in JE, JJ, and SC, but not in GS (Fig. S8).

**Fig. 4:**
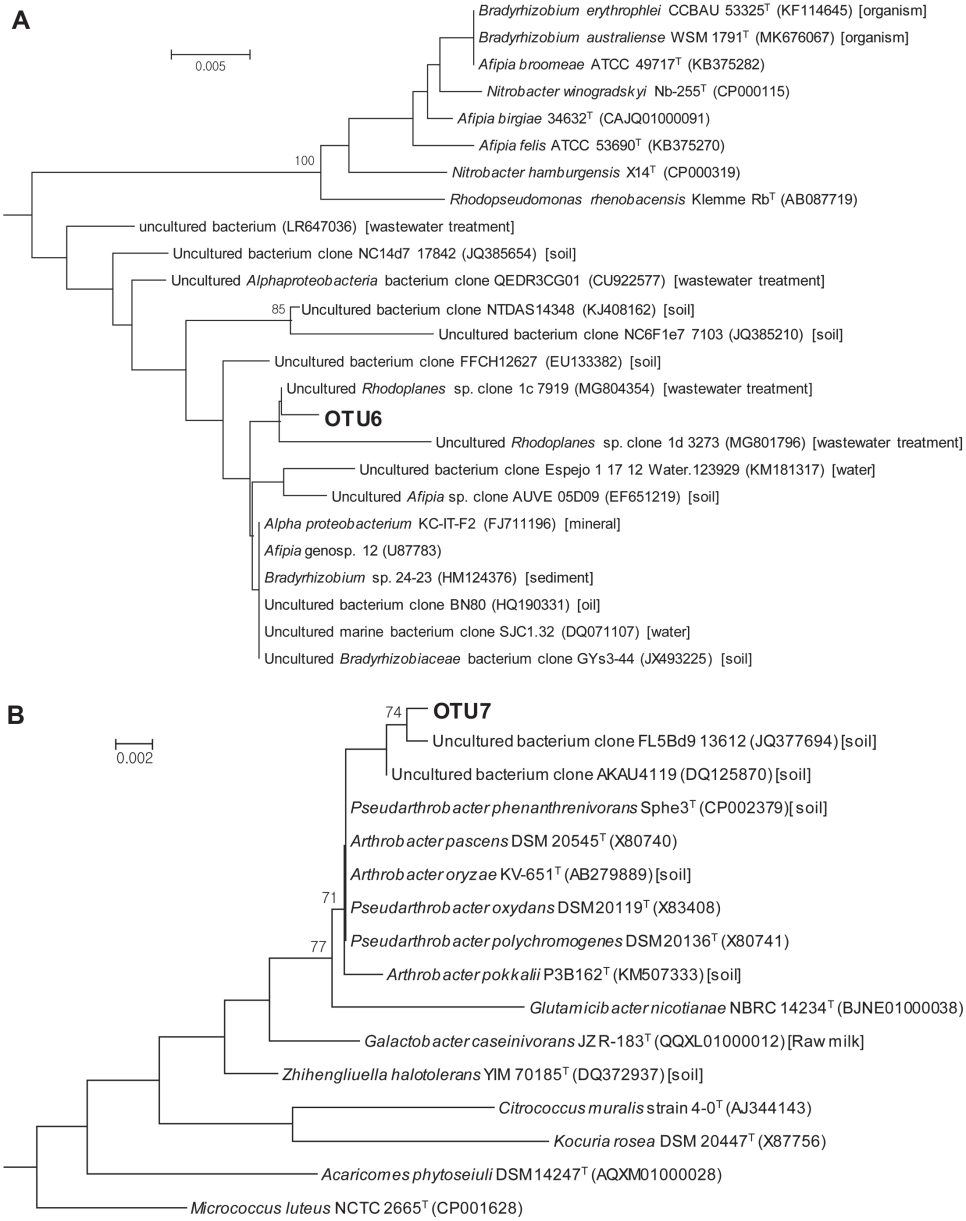
Neighbor-joining tree based on the 16S rRNA gene sequences of the bacterial OTUs enriched on the WPFs (in bold). Bootstra*p* values (> 70%) based on 500 re-samplings are shown at branching points. The scale bars indicate estimated changes per nucleotide. Isolation sources, if known, are indicated in the square brackets.

When a neighbor-joining tree was constructed based on the 16S rRNA gene sequences of OTU6 and the relatives retrieved from GenBank [50] and EzBioCloud [51], OTU6 formed a cluster only with the environmental sequences obtained from metagenomic studies and non-type strains (>97.0% sequence identity) and not with the nearest type strains (<96.0% sequence identity) (Fig. 4A), making it difficult to predict the physiology of OTU6. OTU6 formed a smaller cluster with the environmental sequences obtained from the wastewater treatment system, suggesting that it may be involved in the degradation of pollutants.

In the neighbor-joining tree, OTU7 was clustered with the type strains of the genera *Arthrobacter* and *Pseudarthrobacter* (Fig. 4B), and the latter was formerly classified as *Arthrobacter* [52]. *Arthrobacter* spp. have been reported as a predominant member in many soil habitats, which can be explained by their nutritional versatility and extreme resistance to drying and starvation [53]. They are also known to degrade organic pollutants, such as pesticides, phenolic compounds, phthalates, and aliphatic and polycyclic hydrocarbons [54-56] and produce biosurfactants [57]. Previous studies have shown their involvement in the biodegradation of synthetic polymers, such as polycarbonate [58], nylon oligomers [59], and polyethylene [60, 61]. In addition, recent studies have shown that *Arthrobacter* is one of the enriched bacterial groups on plastics in the terrestrial environment [22, 62]. Based on these previous results, the involvement of OTU7 in the degradation of WPFs is plausible and requires further study.

In a previous study, *Bacillus* was the most enriched bacterial genus (10-50%) on WPFs compared to soils (3.2-9.6%) [20]. Although *Bacillus* did not occupy more than 1.0% on the films for the pulled data of the four landfill sites, it also showed higher abundance on the films than in the soils (Fig. S9), and thus is thought to be one of the bacterial groups adapted to WPFs. This result also indicates that minor groups might be involved in the degradation of plastics, as suggested by MacLean J *et al*. [21], in which the bacterial groups previously known as hydrocarbon degraders, such as *Mycobacterium*, *Burkholderia*, and *Nocardioides*, were enriched on plastic debris but occupied a minor portion of the biofilms.

### Microbial Co-Occurrence Networks in Landfills

**Fig. 5:**
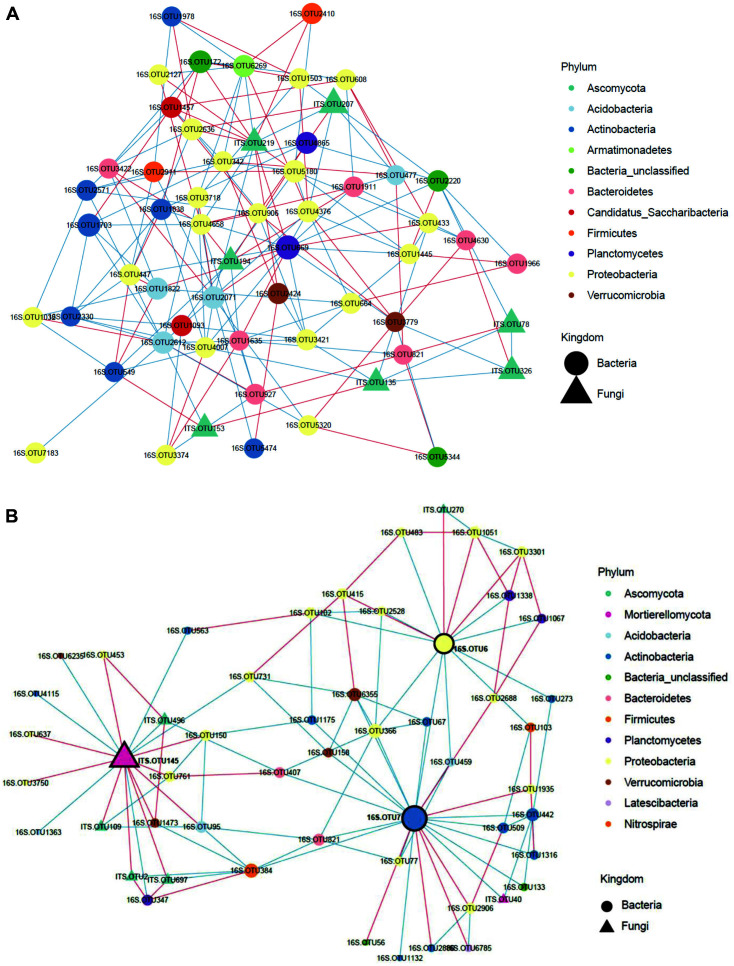
Microbial co-occurrence networks in total landfill samples. The nodes were colored according to the each bacterial and fungal taxonomy at the phylum level. Edge color represents positive (blue) and negative (red) correlations (*p* < 0.01). (**A**) Total co-occurrence network among top tier nodes (degree >35). (**B**) The highlighted co-occurrence network for the film-dominant taxon of 16S.OTU6, 16S.OTU7, and ITS.OTU145.

To identify critical taxa in landfills, we conducted a microbial co-occurrence network analysis and investigated the interaction among bacterial and fungal nodes (Fig. 5). The resulting plastisphere network including bacterial and fungal OTUs consisted of 11,461 edges and 974 nodes which consisted of 904 bacterial nodes and 70 fungal nodes. A total of 904 bacterial nodes belonged to *Proteobacteria* (308 OTUs, 34.1%), *Actinobacteria* (151 OTUs, 16.7%), *Acidobacteria* (139 OTUs, 15.4%), *Bacteroidetes* (74 OTUs, 8.2%) and *Firmicutes* (47 OTUs, 5.2%). 70 fungal nodes consisted of *Ascomycota* (49 OTUs, 70.0%), *Basidomycota* (6 OTUs, 8.6%), *Mortierellomycota* (4 OTUs, 5.7%), and unclassified Fungi (10 OTUs, 14.3%). The average edges per node was 12.1. A total of 11,461 correlated edges consisted of 6,973 positive and 4,524 negative correlations (Fig. 5A).

The top 15 nodes in the inter-domain and bacteria-only network analysis were found not to be the film-dominant taxa (Table S2). This suggests that there was no extensive network among the film-associated taxa. The above-mentioned film-dominant OTUs - bacterial OTU6 (14 edges/node), OTU7 (23 edges/node), and fungal OTU145 (17 edges/node) had more correlations than average (12.1 edges/node), suggesting that they might be the keystone taxa in the degradation of the WPFs. Bacterial OTU6 and OTU7 were directly correlated with each other, but fungal OTU145 was not, suggesting that bacteria and fungi were not directly correlated with each other in the plastisphere (Fig. 5B). Fungal OTU5653, OTU3, and OTU145 of the top 15 keystone OTUs in the fungal-only network were identified as belonging to *Mortierella*, which was reported in a previous study to be a film-colonizing species on the surface of polyethylene films [63], implying that they play an important role in the degradation of this material.

### Functional Prediction of Film-Related Bacterial Communities Using PICRUSt

**Fig. 6:**
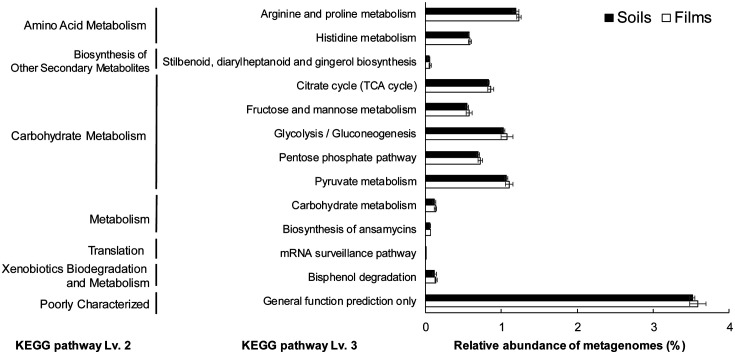
PICRUSt functional prediction using bacterial communities from WPFs and soils. The functions were categorized by KEGG level 2 to 3 and only the functions whose relative abundances were significantly higher in the films than in the soils were represented.

The PICRUSt metagenome prediction was carried out for the functional profiling of film-associated microbiota. The NSTI score of total samples showed a range from 0.12 to 0.23, meaning that the predicted metagenomes were reliable for the following functional study [40]. We categorized the predicted functions by the KEGG pathway level 1 to 3 and identified those with higher relative abundances on the films than in the soils. Increased functions at the KEGG pathway level 3 were amino acid metabolism, carbohydrate metabolism, and xenobiotic biodegradation and metabolism (Fig. 6). Previous studies also observed the increase in the functions related to the metabolisms of carbohydrate and amino acids, and xenobiotic degradation in the plastisphere based on the PICRUSt analysis [20, 64-67].

### Structures of Fungal Communities on WPFs

The PCoA of the fungal communities based on the unweighted (Jaccard) and weighted (Bray-Curtis) dissimilarity indices showed trends similar to those in the bacterial communities (Figs. 2C and 2D). Geographical location was the most important factor determining fungal community structures, and the fungal communities on the films were different from those in the soils. The NMDS analysis of the fungal communities showed similar results to the PCoA analysis even if the stress value of fungi was higher than 0.3 (Fig. S4B). Unlike the PCoA of the bacterial communities (Figs. 2A and 2B), the fungal communities in GS were not separated from the others on the principal coordinate 1. This may be because most fungi are obligate aerobes and the taxonomic structure of the fungal communities would not be significantly changed by the anaerobic condition unlike the bacterial communities in GS, which harbored presumed obligate anaerobes such as *Anaerolineaceae* and *Clostridia* as dominant members (Table S1). In addition, the principal coordinate 1 separated the fungal communities of JJ from the others, supposedly because of the different environment at JJ, that is, forest, compared to the municipal landfills of other sites, which harbored no vegetation, as previously suggested [68, 69].

No fungal phyla or genera were significantly more abundant on the films than in the soils for the pulled data from the four landfill sites (data not shown). At the OTU level, only one OTU, OTU145, was significantly more abundant on the films than in the soils (Fig. 3C). OTU145 was affiliated with the genus *Mortierella*, the majority of which are saprobic soil-inhabiting fungi widespread in temperate zones; it is one of the ten most frequently detected fungal genera in environmental sequencing studies [70]. Several studies have shown that this genus is associated with plastics in oceans [71, 72], and microplastic addition to soil increased its abundance compared to the control treatment [73]. Although there have been no reports on the degradation of plastics by *Mortierella* spp., the consistent enrichment of the fungal genus on plastics in our study and others suggests their potential role in the degradation of plastics. Some authors have designated fungi as potential degraders of environmental plastics [74, 75], and therefore fungi, as well as bacterial communities, should be investigated for their role in plastic degradation.

## Discussion

The present study showed that WPFs buried in landfills for more than 20 years underwent oxidation, although not extensively, and that the microbial communities on these WPFs and in the nearby soils were shaped mainly by geographical location, but were also distinct from each other within the same location. Distinct microbial structures according to geographical location were also observed for the bacterial [76] and fungal communities [77] in the soils of landfills. Song L *et al*. [76] suggested that precipitation and landfill age were the most significant factors driving the bacterial community structure.

It is unclear whether the oxidation of plastic films observed in this study was caused by abiotic or biotic factors. Several microbial groups were enriched on WPFs across the four landfill sites. Among them, members such as *Pseudarthrobacter* and *Mortierella* were also enriched on plastic debris in previous studies, whereas others, such as *Bradyrhizobiaceae*, *Myxococcales*, and *Spartobacteria*, were not.

Two conflicting hypotheses can be drawn from the above results. First, the microbial communities on the WPFs in landfills did not affect the chemical properties of the films and used the films merely as a medium to live on. Second, most organisms on the enriched films were not involved in degradation of the films, but a portion of them was.

It remains unclear whether a core microbiome exists in the plastisphere [23, 78]. For conclusive evidence, long-term laboratory experiments using intact plastics and pure cultures or their consortium suspected of degrading plastic must be performed with the application of omics [79] and instrumental techniques [80] to detect subtle changes in microbial communities and plastics, because the rate of change will be extremely slow. One additional point is that the environmental conditions of landfills may not be favorable for the biodegradation of plastics, considering the present results. This could be due to deficiencies of oxygen and other nutrients, such as nitrogen, in landfills. Thus, other sites, such as composting facilities treating food or livestock waste, to which waste plastics are sometimes introduced as contaminants, need to be considered to screen plastic-degrading microorganisms, as these sites are thought to provide more favorable conditions (*e.g.*, nutrients and oxygen through mixing) for biodegradation of plastics than do landfills [81].

## Supplemental Materials

Supplementary data for this paper are available on-line only at http://jmb.or.kr.

## Figures and Tables

**Table 1:** Description of the four landfill sites.

Location	Gunsan-si, Jeollabuk-do	Jeongeup-si, Jeollabuk-do	Jeju-si, Jeju	Sunchang-gun, Jeollabuk-do
Abbreviation	GS	JE	JJ	SC
GPS	35° 57’ 26” N, 126° 36’ 46” E	35° 36’ 43” N, 126° 51’ 05” E	33° 23’ 4” N, 126° 37’ 15” E	35° 22’ 7” N, 127° 7’ 24” E
Sampling date	2020. 6. 23.	2019. 10. 10.	2019. 5. 27.	2019. 10. 24.
Feature	Landfill site	Landfill site	Mountain forest	Landfill site
Expected minimum period for which the wastes had been buried (years)	26	20	40	28

**Table 2:** Diversity indices of microbial communities.

(A)							

Site	Sample type	Good's coverage	Number of OTUs	Richness estimator		Diversity index	
	
				Chao1	ACE	Shannon	Inverse Simpson

GS	Soil	0.966	1,119	1,638	1,667^a^	4.41	14
	Film	0.980	858	1,118	1,148^b^	4.62	50
JE	Soil	0.932	2,425^a^	3,435^a^	3,438^a^	6.84^a^	321^a^
	Film	0.960	1,576^b^	2,148^b^	2,149^b^	5.91^b^	94^b^
JJ	Soil	0.945	1,954	2,757	2,799	6.34	139
	Film	0.948	1,831	2,598	2,664	6.07	103
SC	Soil	0.943	2,238^b^	2,939	3,009^b^	6.83	428
	Film	0.935	2,431^a^	3,225	3,346^a^	6.80	262

(B)							

Site	Sample type	Good's coverage	Number of OTUs	Richness estimator		Diversity index	
	
				Chao1	ACE	Shannon	Inverse Simpson

GS	Soil	0.999	562^a^	582^a^	582^a^	4.41^a^	29
	Film	0.999	382^b^	405^b^	402^b^	2.78^b^	5
JE	Soil	0.995	1093^a^	1177^a^	1174^a^	5.18^a^	47
	Film	0.999	368^b^	385^b^	386^b^	3.82^b^	19
JJ	Soil	0.998	596	631	618	4.62^a^	32^a^
	Film	0.999	488	521	510	3.63^b^	11^b^
SC	Soil	0.999	578^b^	596^b^	592^b^	4.41	29
	Film	0.997	819^a^	860^a^	854^a^	4.97	51

Species richness and diversities of the bacterial (A) and fungal (B) communities on WPFs and in nearby soils collected from the four landfill sites. All values are the averages of three replicates and different lowercases indicate significant difference between soils and films (*p* < 0.05).

Notes: Number of reads in each sample was normalized to 13,538 for bacteria and 31,919 for fungi.
