# Supplementary material for: Distinct Bacterial and Fungal Communities Colonizing Waste Plastic Films Buried for More Than 20 Years in Four Landfill Sites in Korea
Source: J Microbiol Biotechnol. 2022 Nov 17;32(12):1561–72. doi: 10.4014/jmb.2206.06021 (PMC9843814; doi:10.4014/jmb.2206.06021)
Supplement: Supplementary file 1 [file jmb-32-12-1561-supple.pdf]

J. Microbiol. Biotechnol.
https://doi.org/10.4014/jmb.2206.06021

Supplementary Materials

Distinct bacterial and fungal communities colonizing waste plastic films buried for more than 20 years in four landfill sites in Korea

Joon-hui Chung[1], Jehyeong Yeon[1], Hoon Je Seong[3], Si-Hyun An[1], Da-Yeon Kim[1], Younggun Yoon[1], Hang-Yeon Weon[1], Jeong Jun Kim[1], and Jae-Hyung Ahn[1*]

[1] Agricultural Microbiology Division, National Institute of Agricultural Sciences, Rural Development Administration (RDA), Wanju-gun, Jeollabuk-do, 55365, Republic of Korea

[2] College of Environmental and Bioresource Sciences, Jeonbuk National University, Iksan, 54596, Republic of Korea

[3] Macrogen Inc., Seoul, 06221, Republic of Korea

*

(A) 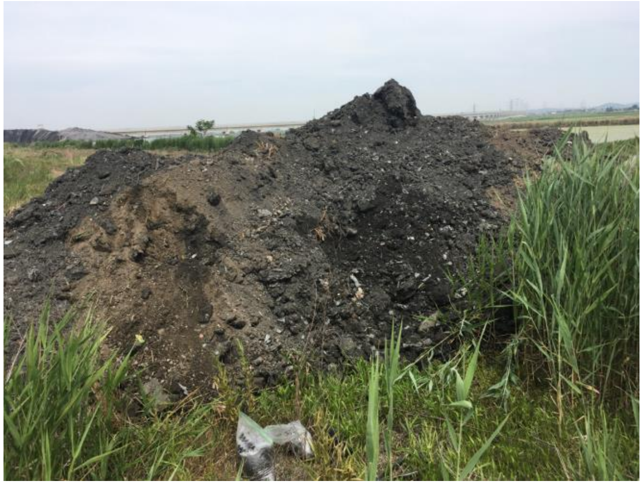 (B) 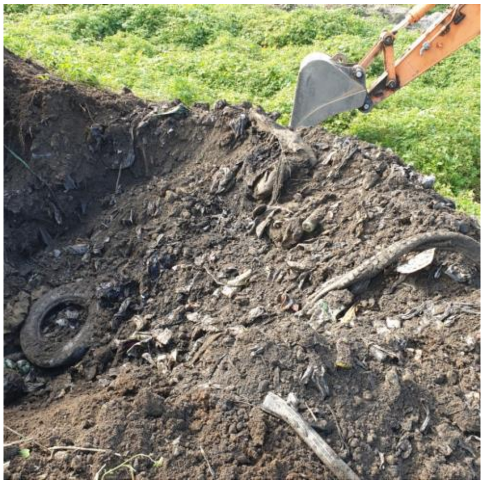

(C)

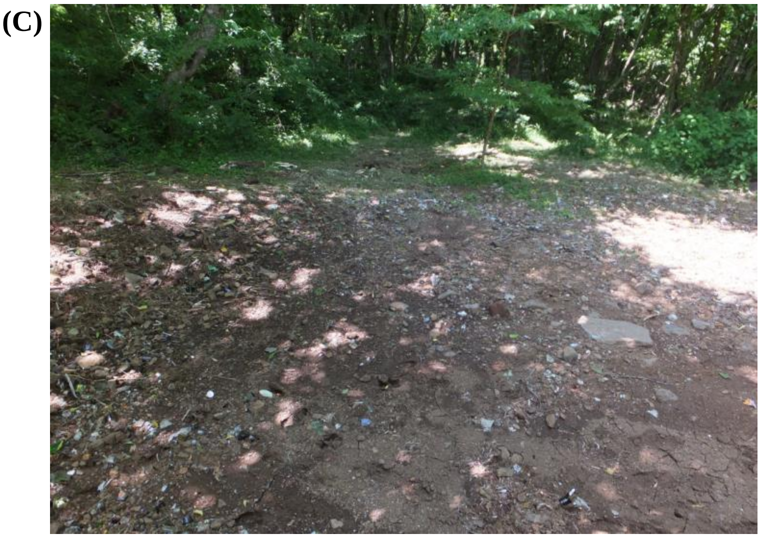

(D)

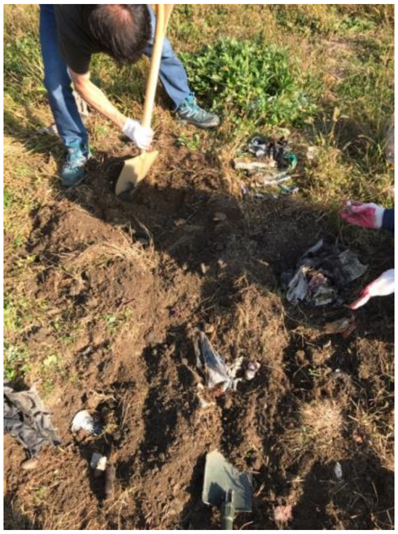

Fig. S1. The four landfill sites from which waste plastic films and nearby soils were collected. (A) Gunsan-si (GS), (B) Jeongeup-si (JE), (C) Jeju-si (JJ), and (D) Sunchang-gun (SC).

(A)

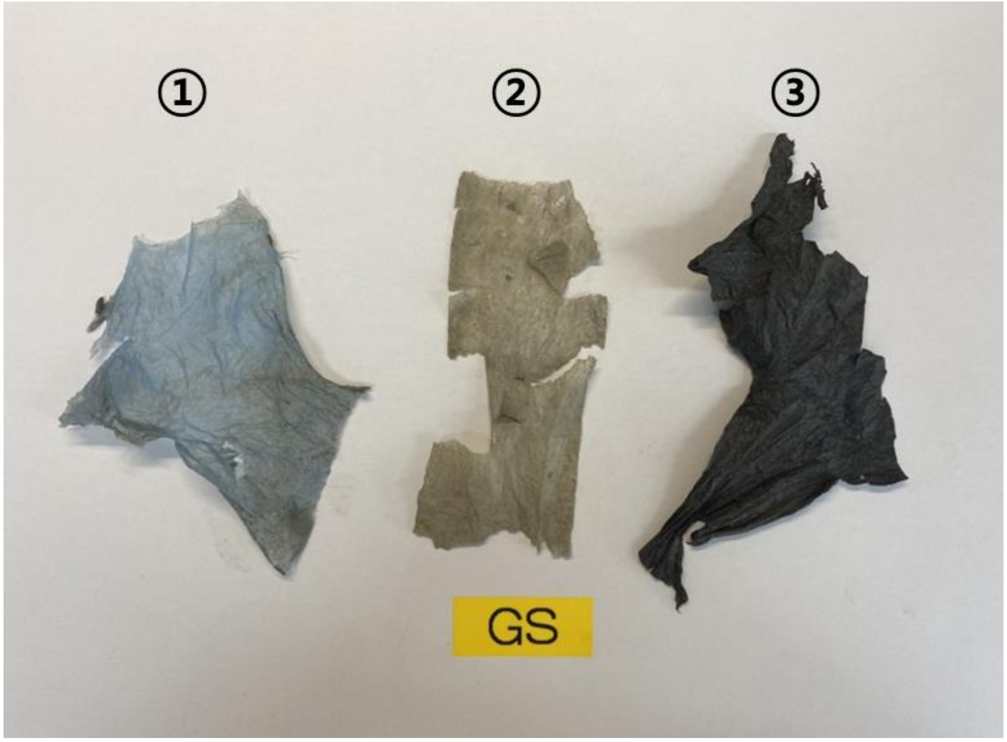

(B)

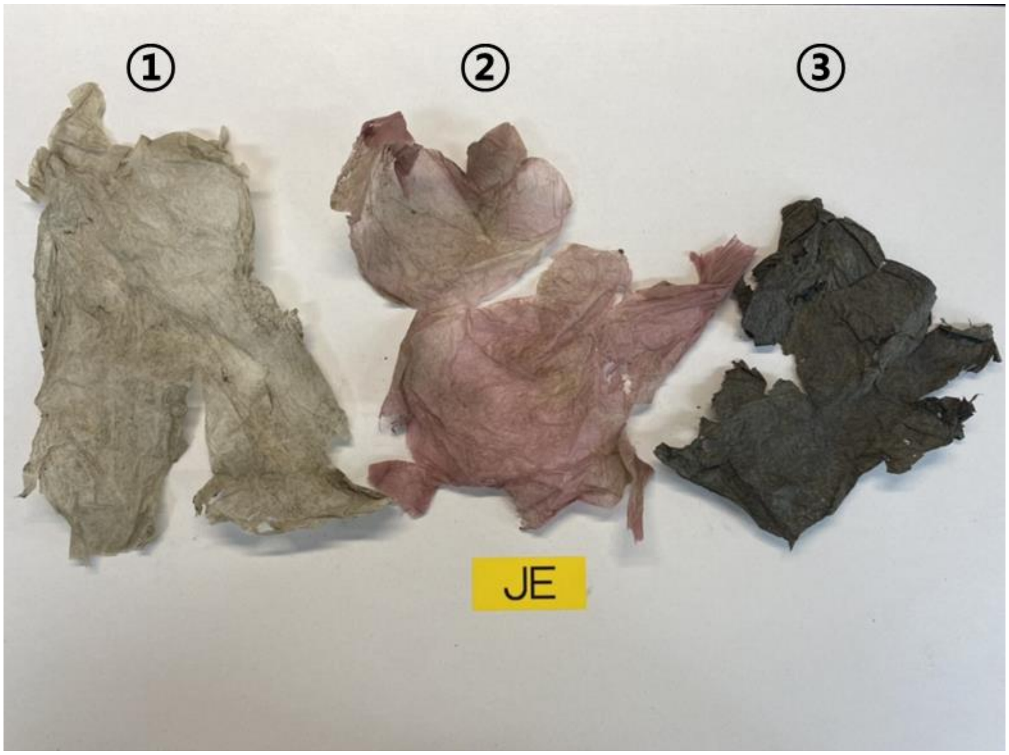

Fig. S2. Waste plastic films collected from the landfill sites of (A) GS, (B) JE, (C) JJ, and (D) SC.

(C)

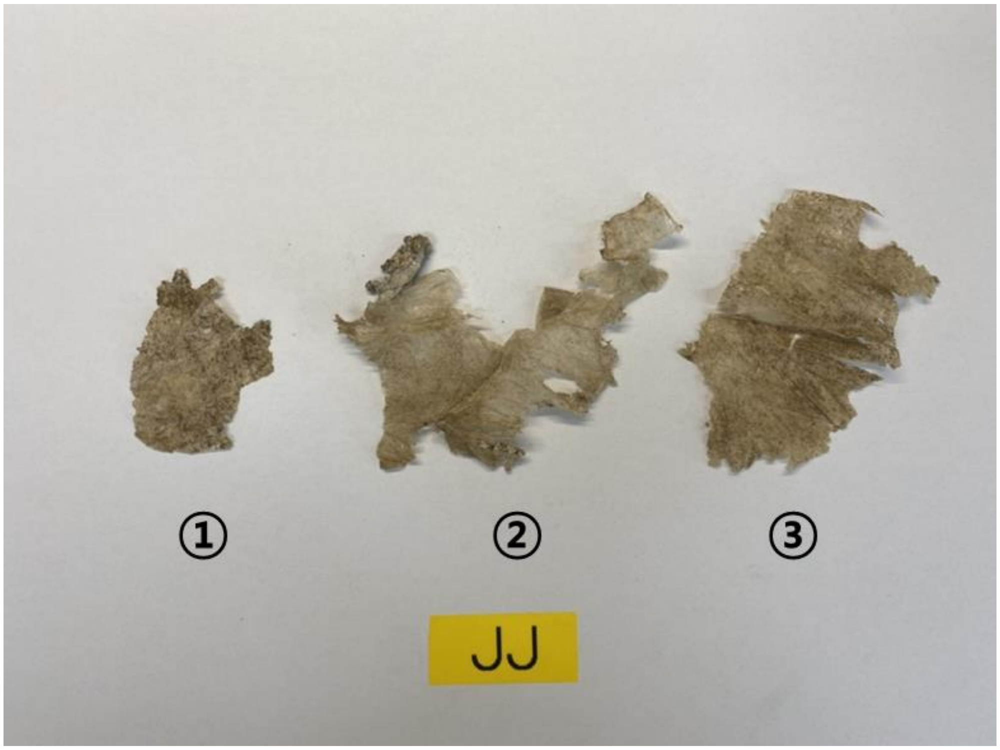

(D)

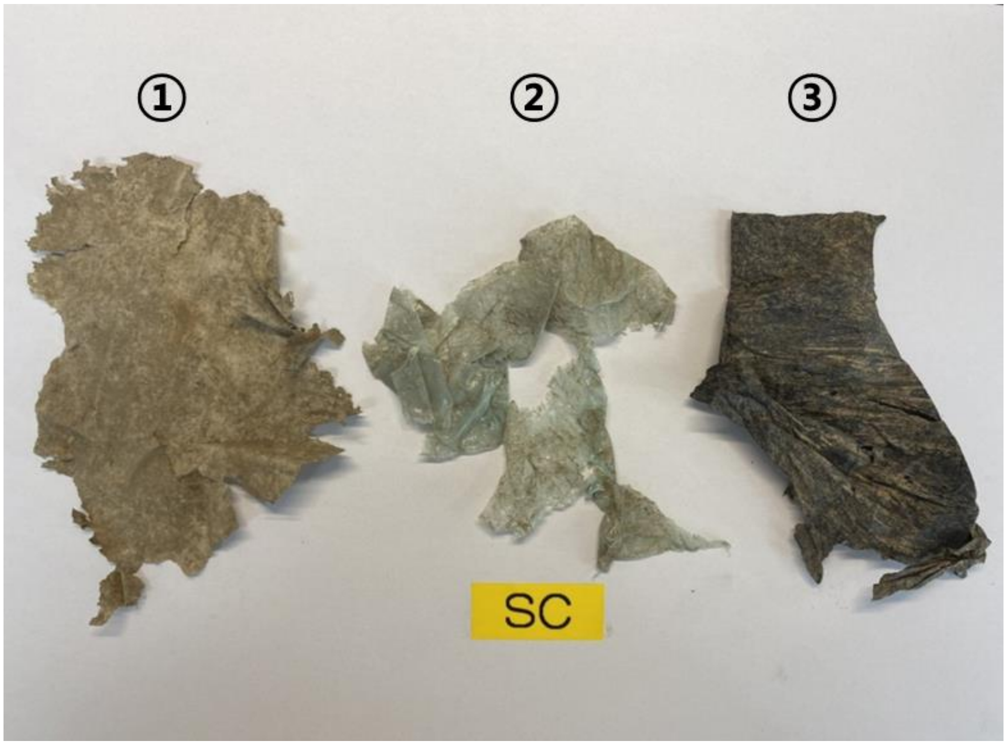

Fig. S2. *Continued*.

(A)

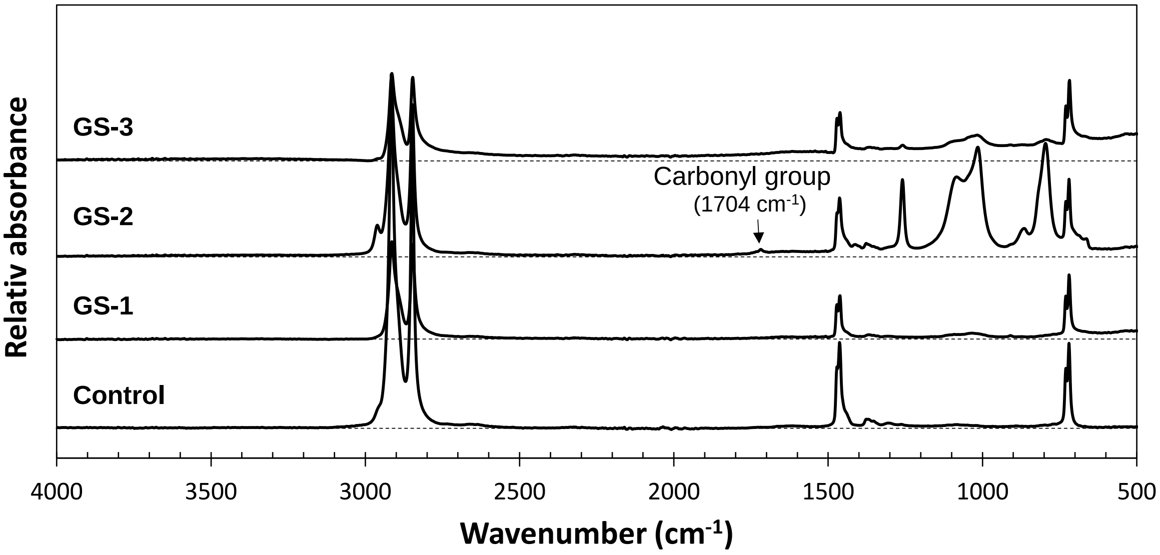

(B)

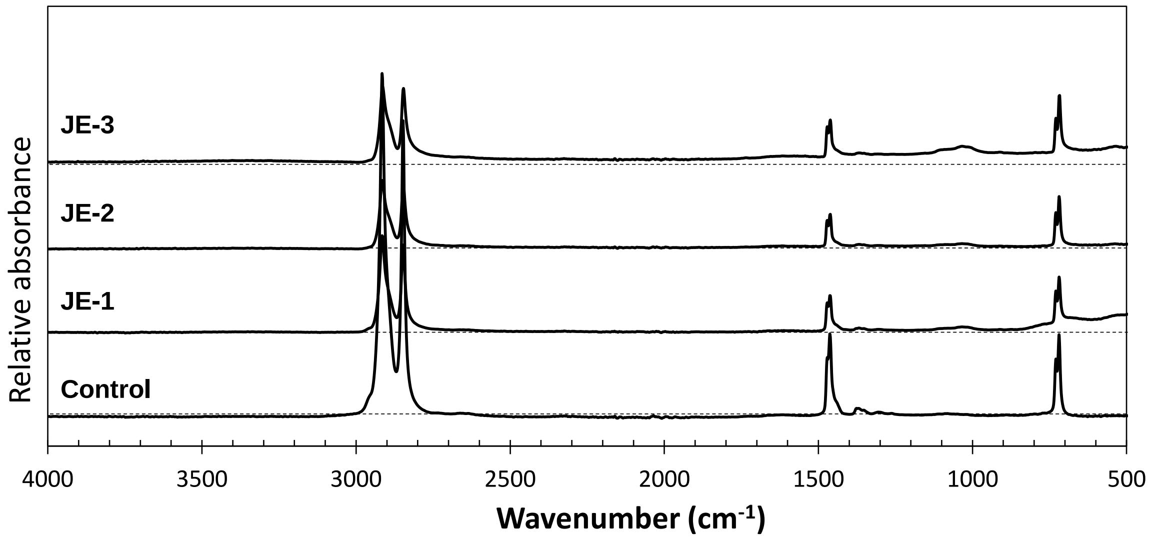

Fig. S3. Infrared spectra of the waste plastic films collected from the landfill sites of (A) GS, (B) JE, (C) JJ, and (D) SC. Three different waste plastic films were analyzed for each site. The spectrum of the control was obtained using an additive-free LDPE film.

(C)

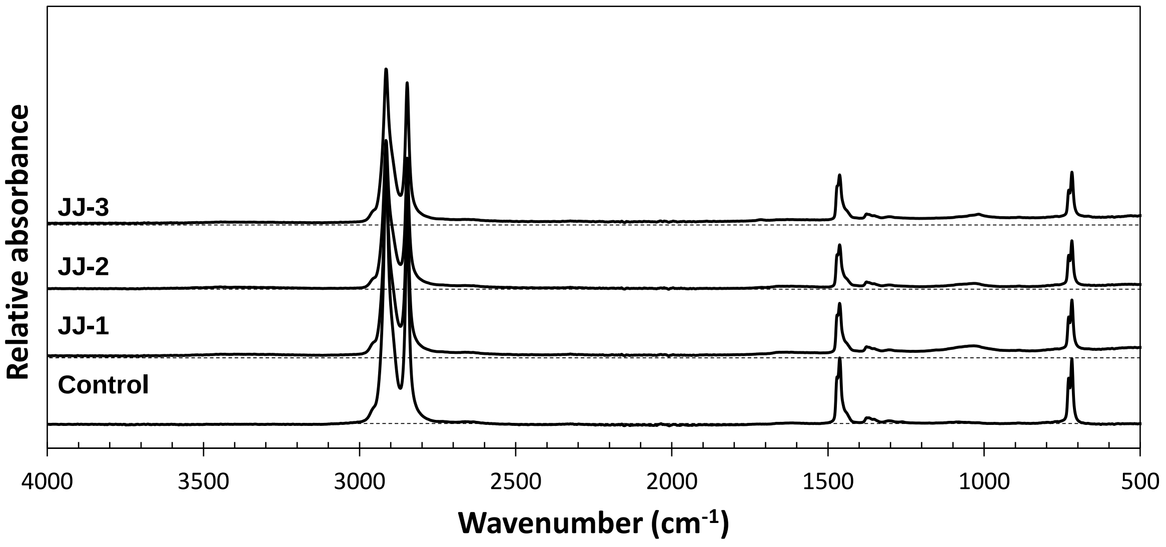

(D)

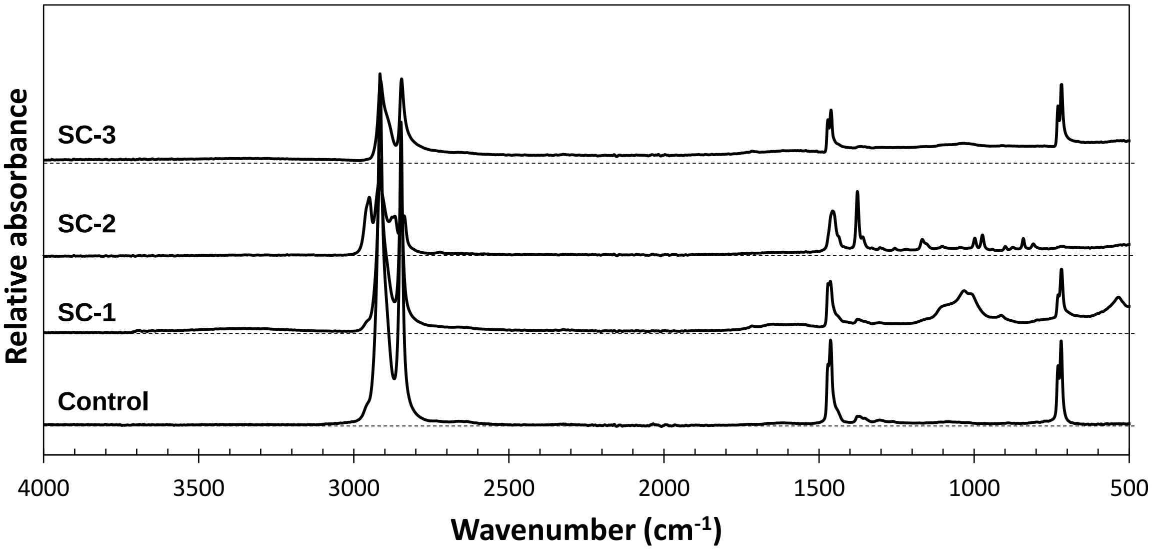

Fig. S3. *Continued.*

(A)

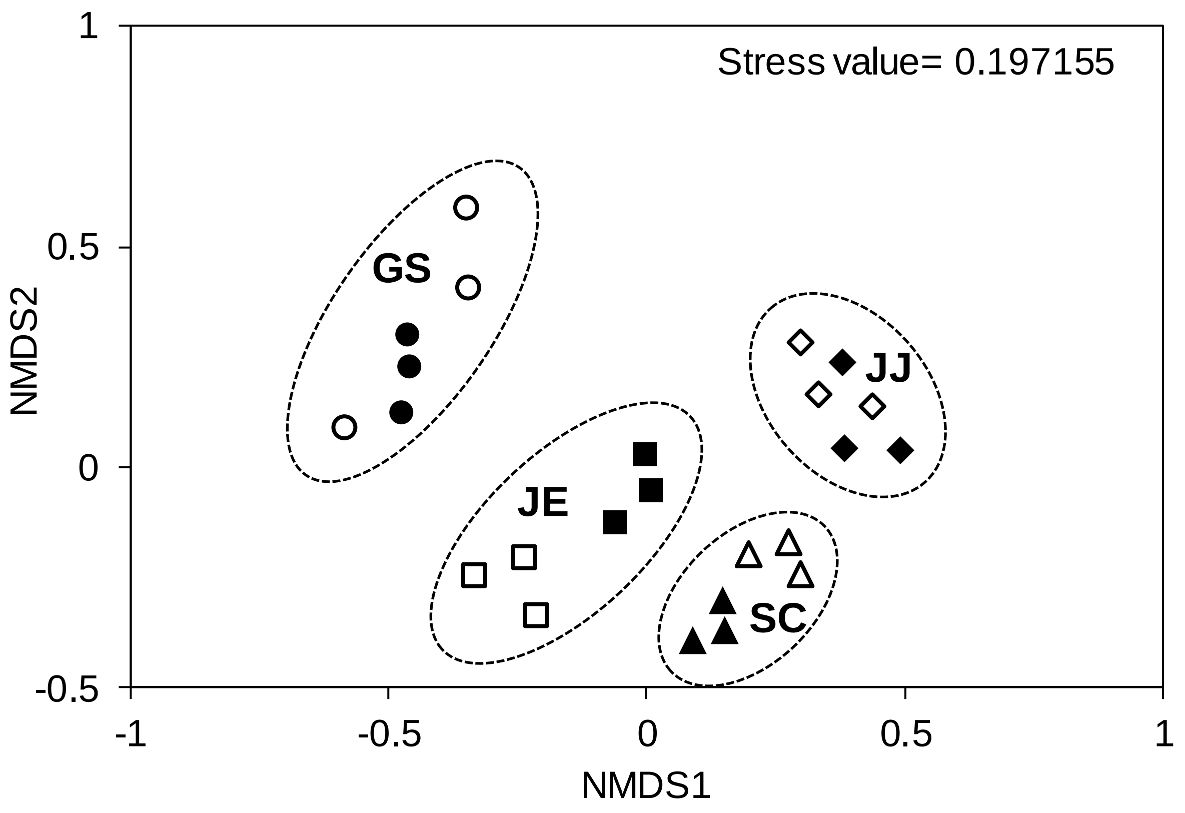

Fig. S4. NMDS analysis of the microbial communities. Bacterial (A) and fungal (B) communities on the waste plastic films (open symbols) and in the nearby soils (closed symbols) were analysed with the Sørenson distances.

(B)

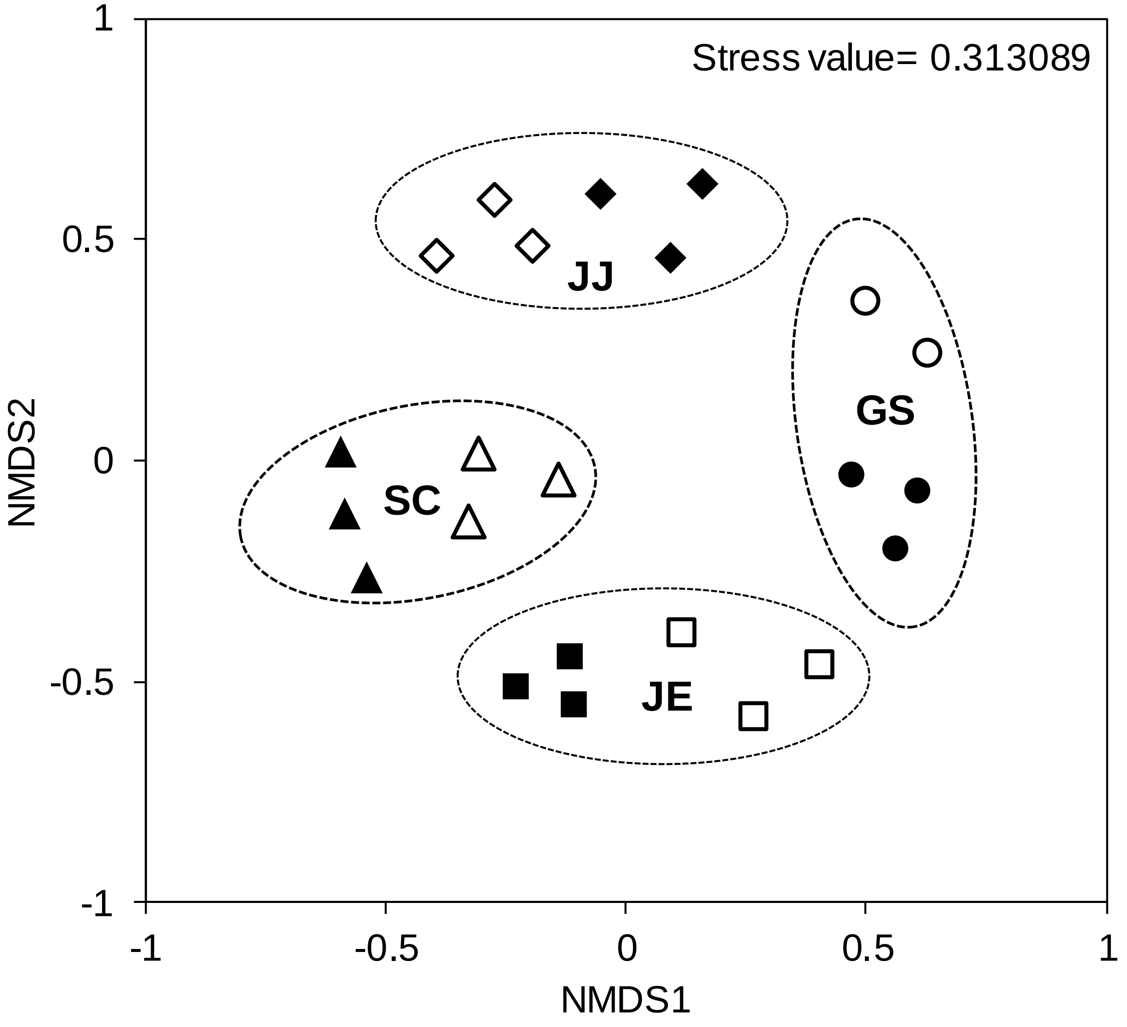

Fig. S4. *Continued.*

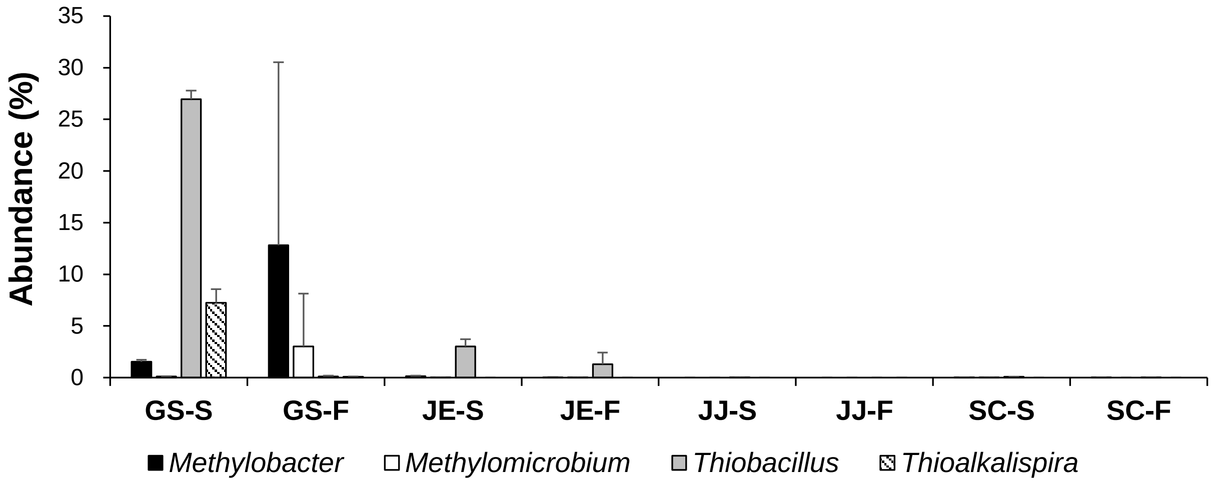

Fig. S5. The relative abundances of methane-oxidizing and sulfur-oxidizing bacteria among landfill environment. The relative abundances of methane-oxidizing (*Methylobacter* and *Methylomicrobium*) and sulfur-oxidizing (*Thiobacillus* and *Thioalkalispira*) bacteria among the bacterial communities in the nearby soils (-S) and on the waste plastic films (-F) were shown and collected from the four landfill sites.

(A)

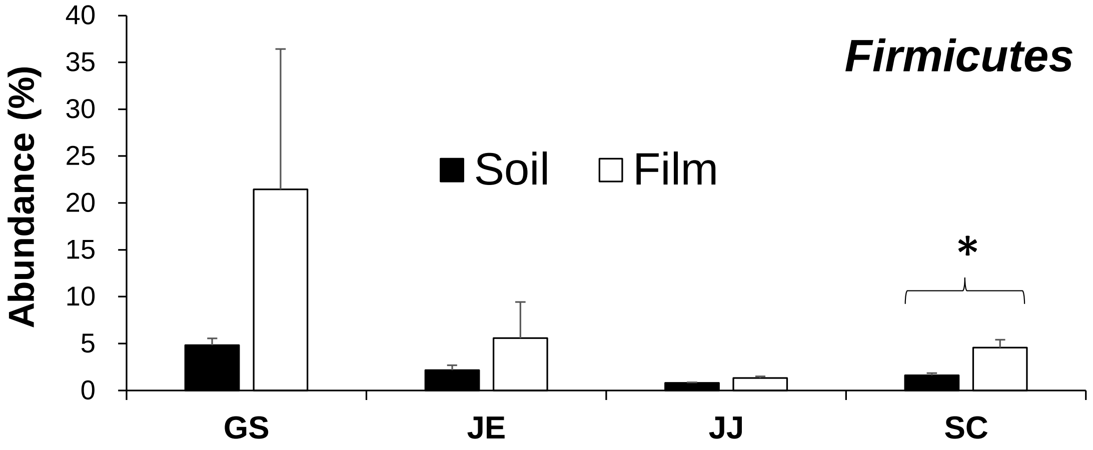

(B)

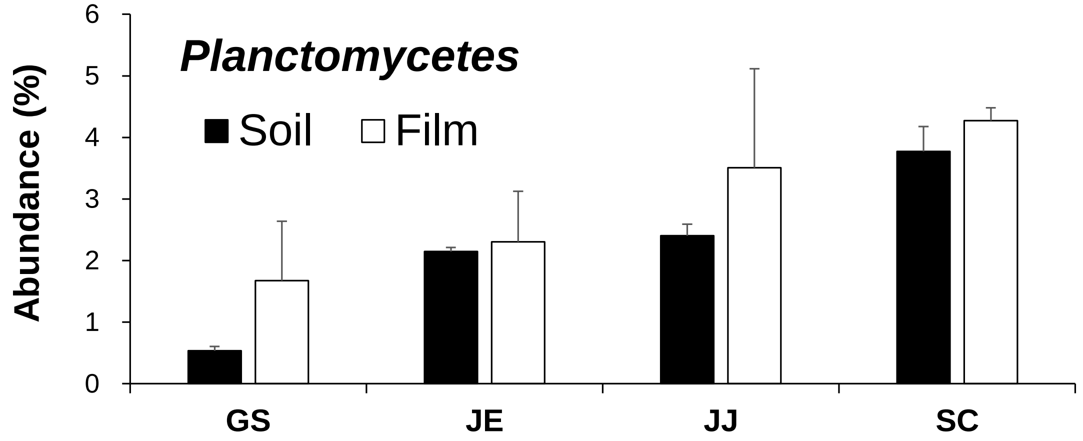

(C)

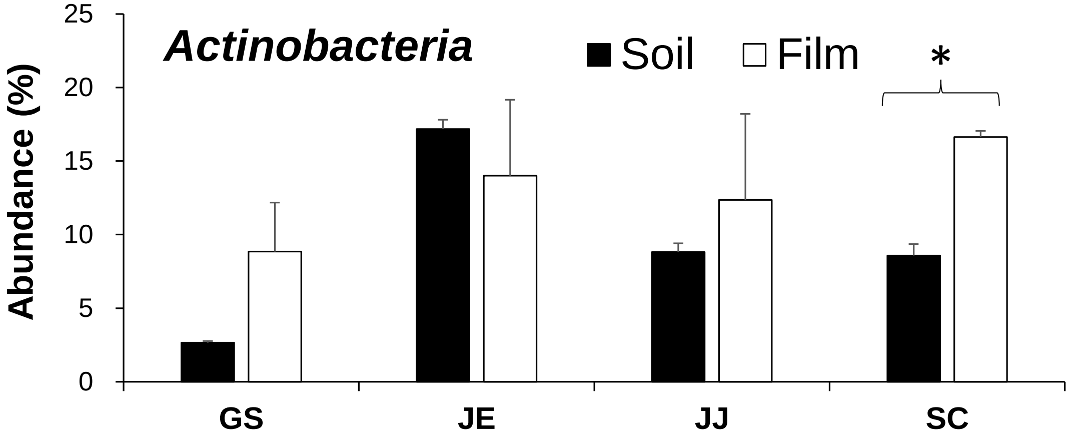

Fig. S6. The relative abundances of the film-enriched bacterial phyla in the four landfill sites. The values are means ± standard deviations, *n*=3. The asterisks (*) indicate that the abundance on the film is significantly higher than that in the soil ($P<0.05$).

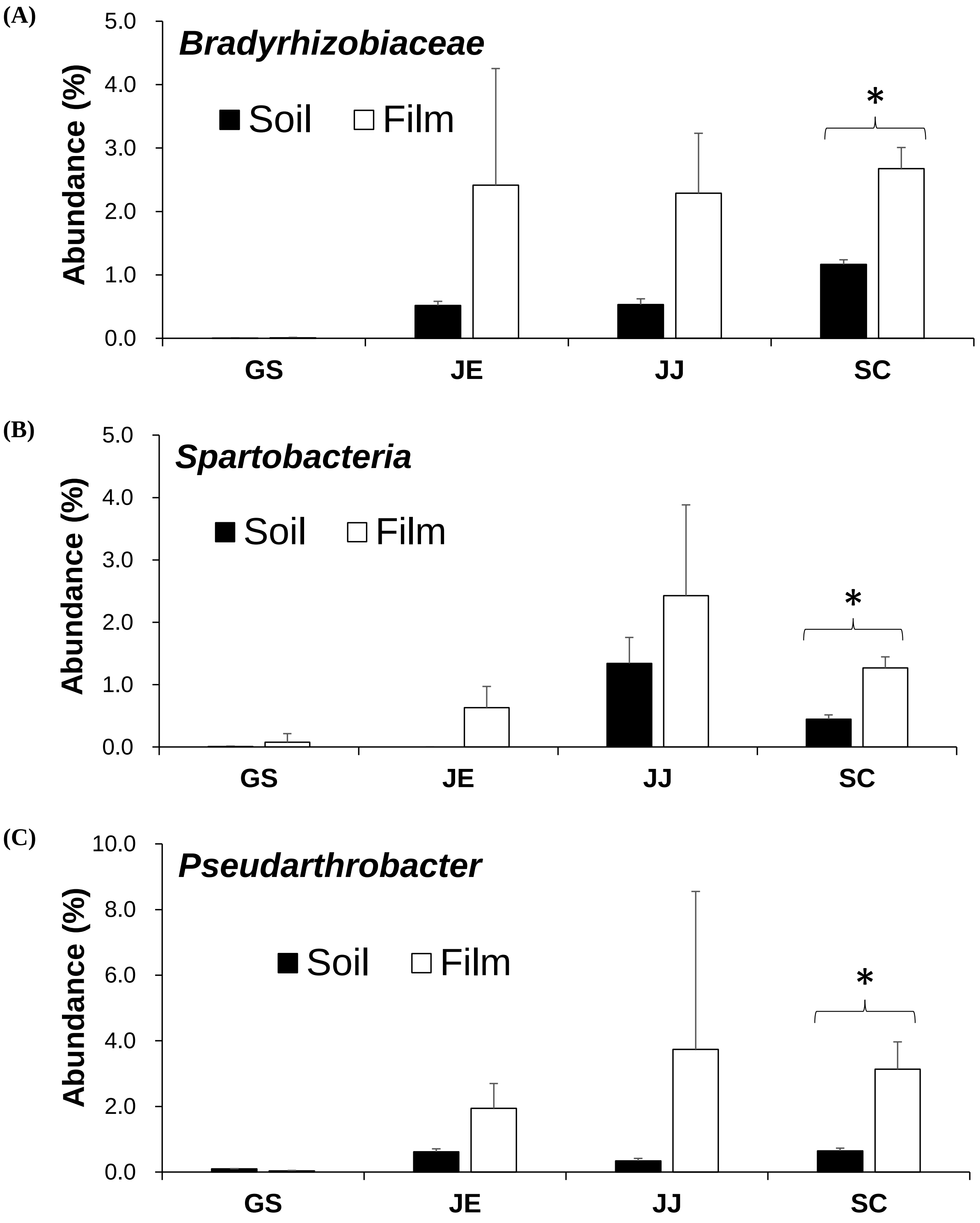

Fig. S7. The relative abundances of the film-enriched bacterial genus in the four landfill sites. The values are means ± standard deviations, *n*=3. The asterisks (*) indicate that the abundance on the film is significantly higher than that in the soil (P<0.05).

(D)
(E)
(F)

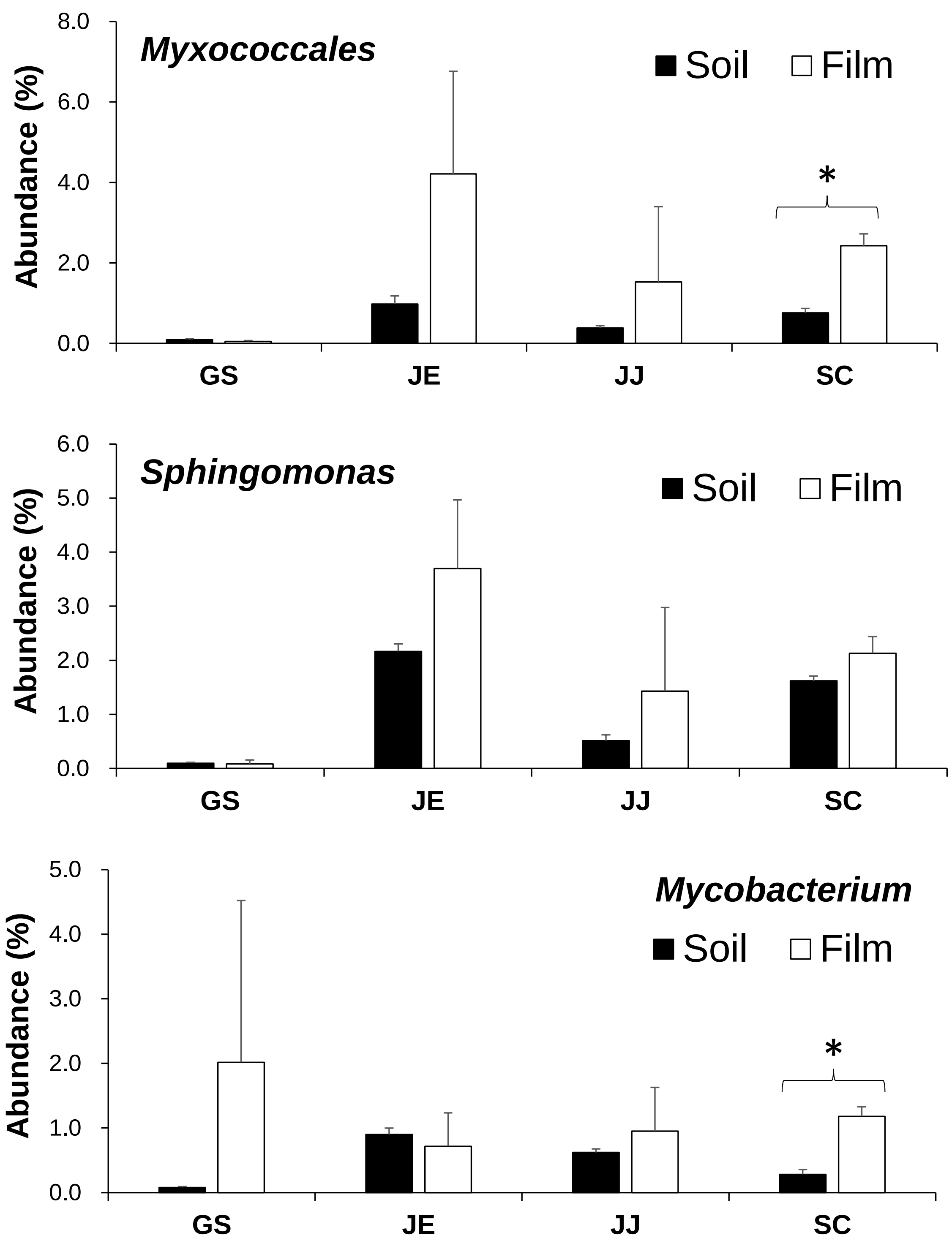

Fig. S7. *Continued.*

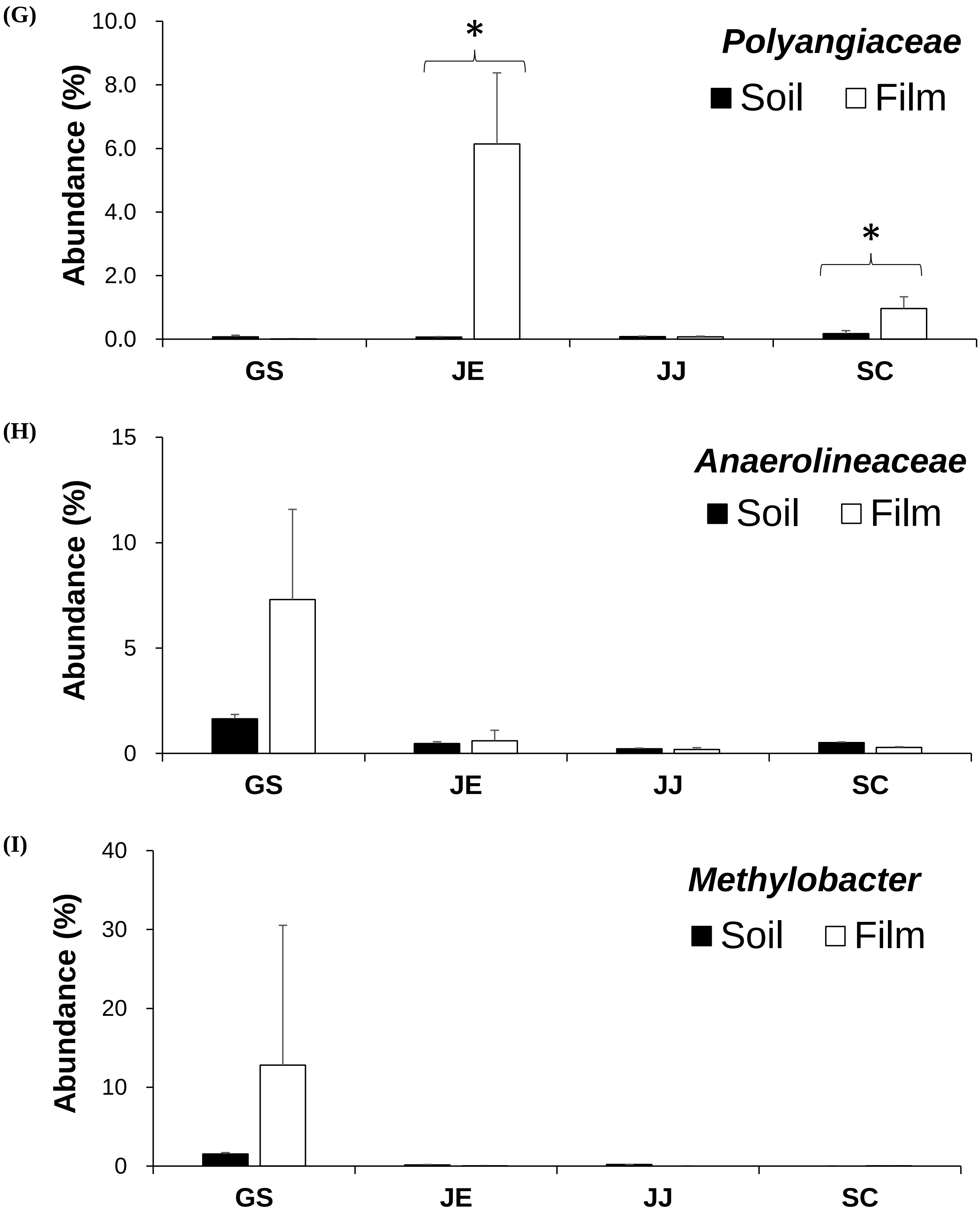

Fig. S7. *Continued.*

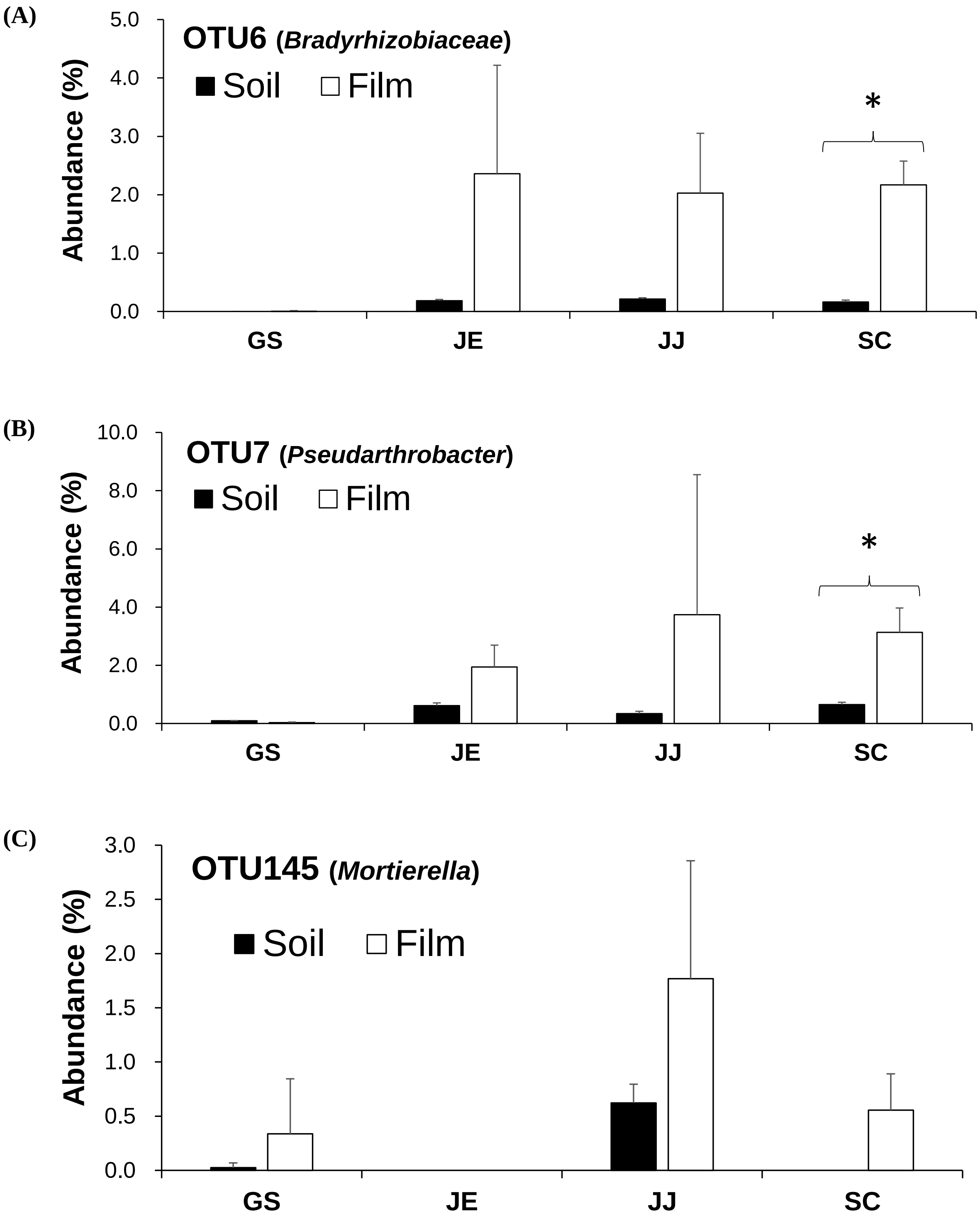

Fig. S8. The relative abundances of the film-enriched bacterial species in the four landfill sites. The values are means ± standard deviations, *n*=3. The asterisks (*) indicate that the abundance on the film is significantly higher than that in the soil ($P<0.05$).

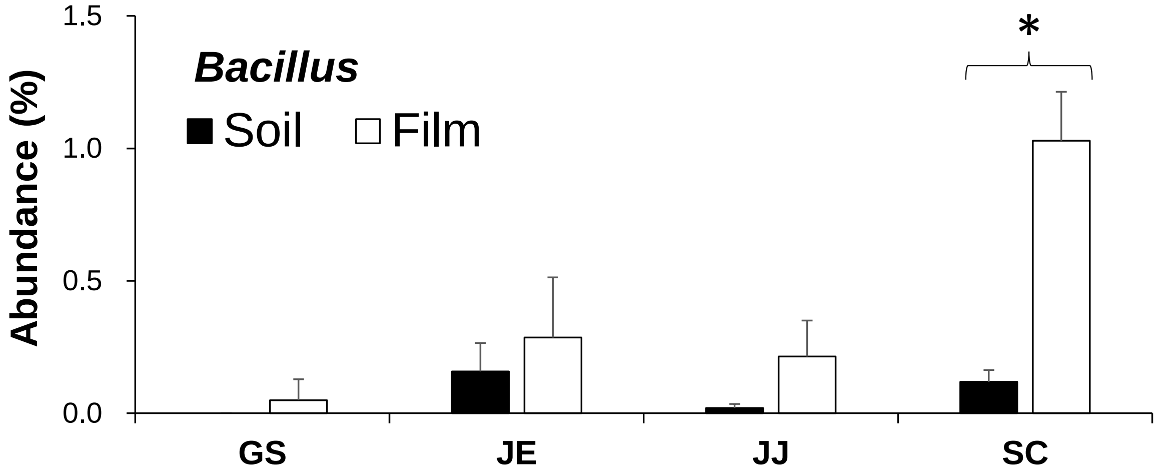

Fig. S9. The relative abundances of *Bacillus* in the soils and on the films in the four landfill sites. The values are means ± standard deviations, n=3. The asterisks (*) indicate that the abundance on the film is significantly higher than that in the soil (P<0.05).

Supplementary table S2. The top 15 nodes from co-occurrence network of landfill plastisphere

The top 15 nodes of the operational taxonomic units (OTUs) in each microbial community on the waste plastic films (F) and in the nearby soils (S) collected from Gunsan-si (GS), Jeongeup-si (JE), Jeju-si (JJ), and Sunchang-gun (SC).

The data in a column was retrieved from one sample among the three replicates.

**Bacterial and fungal top 15 nodes**										
node	n	Taxonomy	GSF	GSS	JEF	JES	JJF	JJS	SCF	SCS
16S.OTU669	51	Gemmata	0.00	0.00	0.01	0.02	0.01	0.01	0.03	0.03
16S.OTU1703	47	Solirubrobacter	0.00	0.00	0.01	0.01	0.01	0.01	0.01	0.02
16S.OTU172	45	Bacteria_unclassified	0.00	0.00	0.01	0.02	0.14	0.03	0.03	0.00
16S.OTU5180	45	Polyangiaceae_unclassified	0.00	0.00	0.00	0.01	0.01	0.00	0.03	0.04
16S.OTU2071	44	c_Gp16_unclassified	0.00	0.00	0.02	0.03	0.03	0.03	0.03	0.04
16S.OTU2612	44	c_Gp1_unclassified	0.00	0.00	0.00	0.02	0.01	0.01	0.01	0.01
16S.OTU1822	43	c_Gp16_unclassified	0.01	0.01	0.01	0.02	0.01	0.03	0.02	0.02
16S.OTU6269	43	c_Armatimonadetes_gp5_unclassified	0.00	0.00	0.00	0.03	0.01	0.01	0.02	0.02
ITS.OTU207	43	Paraphoma	0.00	0.00	0.02	0.06	0.01	0.07	0.14	0.09
16S.OTU2636	42	f_Hyphomicrobiaceae_unclassified	0.00	0.00	0.02	0.02	0.00	0.00	0.01	0.01
16S.OTU4376	42	Acidibacter_unclassified	0.00	0.00	0.00	0.00	0.01	0.01	0.01	0.01
16S.OTU4865	42	Pirellula	0.00	0.00	0.00	0.03	0.03	0.02	0.02	0.02
16S.OTU2220	40	Bacteria_unclassified	0.00	0.00	0.01	0.01	0.02	0.00	0.03	0.01
16S.OTU3718	40	c_Betaproteobacteria_unclassified	0.00	0.00	0.01	0.01	0.00	0.00	0.01	0.02
16S.OTU447	40	Duganella	0.00	0.00	0.00	0.15	0.01	0.05	0.01	0.03
**Bacterial top 15 nodes**										
node	n	Bacterial taxonomy	GSF	GSS	JEF	JES	JJF	JJS	SCF	SCS
OTU669	48	Gemmata	0.00	0.00	0.01	0.02	0.01	0.01	0.03	0.03
OTU6269	42	c_Armatimonadetes_gp5_unclassified	0.00	0.00	0.00	0.03	0.01	0.01	0.02	0.02
OTU1838	39	Gaiella	0.00	0.00	0.00	0.01	0.01	0.02	0.03	0.03
OTU4865	39	Pirellula	0.00	0.00	0.00	0.03	0.03	0.02	0.02	0.02
OTU1457	37	p_Candidatus_Saccharibacteria_unclassified	0.00	0.00	0.03	0.02	0.01	0.02	0.01	0.01
OTU2636	37	f_Hyphomicrobiaceae_unclassified	0.00	0.00	0.02	0.02	0.00	0.00	0.01	0.01
OTU2071	36	c_Gp16_unclassified	0.00	0.00	0.02	0.03	0.03	0.03	0.03	0.04
OTU2612	36	c_Gp1_unclassified	0.00	0.00	0.00	0.02	0.01	0.01	0.01	0.01
OTU8813	36	f_Micromonosporaceae_unclassified	0.04	0.01	0.05	0.05	0.02	0.02	0.04	0.10
OTU1703	35	Solirubrobacter	0.00	0.00	0.01	0.01	0.01	0.01	0.01	0.02
OTU1803	35	c_Gp6_unclassified	0.00	0.00	0.01	0.03	0.01	0.00	0.01	0.02
OTU1978	35	f_Iamiaceae_unclassified	0.00	0.00	0.01	0.02	0.01	0.05	0.01	0.01
OTU406	35	Pseudomonas	0.16	0.06	0.03	0.07	0.26	0.13	0.07	0.06
OTU4658	35	Aquincola	0.00	0.00	0.02	0.01	0.07	0.02	0.07	0.03
OTU664	35	Dokdonella	0.00	0.01	0.07	0.17	0.02	0.04	0.08	0.01
**Fungal top 15 nodes**										
node	n	fungal taxonomy	GSF	GSS	JEF	JES	JJF	JJS	SCF	SCS
OTU74	6	p_Ascomycota_unclassified	0.01	0.00	0.00	0.07	0.26	0.36	0.46	0.30
OTU134	4	Staphylotrichum	0.00	0.00	0.00	0.02	0.33	0.42	0.00	0.02
OTU31	4	Fusarium	0.05	0.13	1.73	1.49	0.00	0.00	0.16	0.41
OTU5653	4	Mortierella	0.00	0.00	0.00	0.02	0.35	0.57	0.77	0.04
OTU1	3	o_Sordariales_unclassified	0.00	0.00	18.49	0.88	0.00	0.00	0.20	0.17
OTU22	3	Pseudopithomyces	0.00	0.00	0.78	2.08	0.00	0.00	0.50	0.51
OTU254	3	f_Cordycipitaceae_unclassified	0.00	0.00	0.01	0.01	0.23	0.14	0.09	0.00
OTU3	3	Mortierella	0.00	0.00	0.32	1.93	8.36	3.84	1.72	0.01
OTU349	3	Trichoderma	0.00	0.00	0.05	0.30	0.05	0.11	0.07	0.02
OTU496	3	Tolypocladium	0.00	0.00	0.00	0.00	0.04	0.05	0.06	0.00
OTU67	3	Gibberella	0.01	0.02	0.01	0.05	0.47	1.07	0.00	0.09
OTU116	2	Cadophora	0.00	0.02	0.00	0.29	0.15	0.57	0.22	0.00
OTU119	2	Fungi_unclassified	0.00	0.00	0.35	0.19	0.02	0.01	0.87	0.16
OTU124	2	Metarhizium	0.00	0.06	0.04	0.47	0.29	0.29	0.00	0.09
OTU145	2	Mortierella	0.34	0.03	0.00	0.00	1.77	0.62	0.56	0.00
